# A cause–effect relationship between Graves’ disease and the gut microbiome contributes to the thyroid–gut axis: A bidirectional two-sample Mendelian randomization study

**DOI:** 10.3389/fimmu.2023.977587

**Published:** 2023-02-14

**Authors:** Jiamin Cao, Nuo Wang, Yong Luo, Chen Ma, Zhuokun Chen, Changci Chenzhao, Feng Zhang, Xin Qi, Wei Xiong

**Affiliations:** ^1^ Department of Ophthalmology, The Third Xiangya Hospital, Central South University, Hunan, China; ^2^ Department of Ophthalmology, The Second Xiangya Hospital, Central South University, Hunan, China

**Keywords:** Graves’ disease, gut microbiome, causal effect, Mendelian randomization, thyroid–gut-axis

## Abstract

**Background:**

An association between Graves’ disease (GD) and the gut microbiome has been identified, but the causal effect between them remains unclear.

**Methods:**

Bidirectional two-sample Mendelian randomization (MR) analysis was used to detect the causal effect between GD and the gut microbiome. Gut microbiome data were derived from samples from a range of different ethnicities (18,340 samples) and data on GD were obtained from samples of Asian ethnicity (212,453 samples). Single nucleotide polymorphisms (SNPs) were selected as instrumental variables according to different criteria. They were used to evaluate the causal effect between exposures and outcomes through inverse-variance weighting (IVW), weighted median, weighted mode, MR-Egger, and simple mode methods. *F*-statistics and sensitivity analyses were performed to evaluate bias and reliability.

**Results:**

In total, 1,560 instrumental variables were extracted from the gut microbiome data (*p*< 1 × 10^5^). The classes *Deltaproteobacteria* [odds ratio (OR) = 3.603] and *Mollicutes*, as well as the genera *Ruminococcus torques* group, *Oxalobacter*, and *Ruminococcaceae* UCG 011 were identified as risk factors for GD. The family *Peptococcaceae* and the genus *Anaerostipes* (OR = 0.489) were protective factors for GD. In addition, 13 instrumental variables were extracted from GD (*p*< 1 × 10^–8^), causing one family and eight genera to be regulated. The genus *Clostridium innocuum* group (*p* = 0.024, OR = 0.918) and *Anaerofilum* (*p* = 0.049, OR = 1.584) had the greatest probability of being regulated. Significant bias, heterogeneity, and horizontal pleiotropy were not detected.

**Conclusion:**

A causal effect relationship exists between GD and the gut microbiome, demonstrating regulatory activity and interactions, and thus providing evidence supporting the involvement of a thyroid–gut axis.

## Introduction

Graves’ disease (GD) is an autoimmune disease with a lifetime risk of 3% for women and 0.5% for men (1). The classic manifestations of GD include hyperthyroidism, palpitations, and ophthalmopathy (2). Although GD has been recognized for over two centuries, the underlying pathological mechanisms have not been fully elucidated. Many risk factors have been suggested to be involved in the disease process, such as genetics, epigenetics, smoking, stress, the gut microbiome, and infection (3). GD is an immune-related disease, with many up-regulated microRNAs, proteins, antibodies, and cytokines present in the circulatory system, but the signaling mechanisms between risk factors and dysfunction of the immune system remain unclear (4). Based on the bio-psycho-social matrix model, integrated research has been used to detect the pathogenesis of GD and other risk factors involved in GD, such as social stress (3, 5). Research on the association between GD and other organs has also been performed.

The concept of the thyroid–gut axis (TGA) has been used to explore the association between the thyroid gland and the gut, and the intestinal influence on the thyroid has been described as involving the uptake of micronutrients and regulation of immune responses (6). In recent years, with the development of sequencing technology, the mechanisms utilized by the gut microbiome in regulating disease processes have been elucidated, indicating that the gut microbiome regulates the immune response and metabolism as well as interactions with drugs (7, 8). Many studies have identified a relationship between the gut microbiome and the thyroid gland (9). The gut microbiome is associated with the incidence of GD, as indicated by the variation in gut microbiome composition observed between participants with GD and participants without GD (9). Recent basic research has illustrated that the gut microbiome may be involved in the loss of self-tolerance and auto-aggressive damage, thus participating in the pathogenesis of GD (9). Covelli and Ludgate (10) concluded that, because the gut provides the first and the widest exposure to bacteria, any change in the environment may influence the microbial balance. This may further activate the gut immune system, including the activation of Treg and Th17 cells, promoting the incidence of GD (10). Clinical research has identified direct changes in the gut microbiome in GD. A study involving 55 participants with GD and 48 without suggested that the gut microbiome differed significantly between the two groups, with the levels of 18 taxa increased and four taxa decreased in those with GD (11). A study by Ei-Zawawy et al. revealed that the *Firmicutes*-to-*Bacteroidetes* ratio was decreased in individuals with GD, which suggested that the gut microbiome may participate in the pathogenesis of GD (12). However, the generalization of the findings or ability to make a definitive conclusion was limited owing to the small number of fecal samples evaluated (13). In addition to clinical research, basic medical research has also provided evidence on the TGA. For example, a murine model of GD was used to compare the gut microbiome with control models, and the results showed that levels of *Firmicutes* were significantly positively related to orbital adipogenesis (14).

A relationship between the gut microbiome and GD has been identified, but a causal effect is unclear. A causal effect is difficult to identify through clinical trials owing to ethical issues and costs. Many studies investigating the association between the gut microbiome and GD are case–control studies, thus making it difficult to determine whether changes in the composition of the gut microbiome or the onset of GD occurred first (11, 15, 16). Mendelian randomization (MR) is a method that uses single nucleotide polymorphisms (SNPs) as an instrumental variable (IV) from a genome-wide association study (GWAS) to detect the causal effect between exposure and outcome (17). SNPs are defined by a variation of a single nucleotide in a specific position of a DNA sequence (18). As SNPs are randomized and allocated genetically to individuals before exposure and outcome and after excluding the effect of linkage disequilibrium, many confounders acquired after zygote formation can be excluded when using SNPs as an IV (19). Research using SNPs as IVs has increased in recent years (19). In addition, as SNPs are related to both exposure and outcomes, SNPs extracted from exposure were instrumental variables linking exposure and outcome. By this process, SNPs have been used to group exposure and non-exposure to evaluate the causal effect of exposure on the outcome, similar to the randomized controlled trial (20). For example, a causal effect has been detected between fat composition and type 2 diabetes (T2D) through MR (21).

For the present study, GWAS summary datasets of the gut microbiome (18,340 samples) and GD (212,453 samples) were obtained from the international consortium MiBioGen and BioBank Japan, respectively (22, 23). Both GWAS summary datasets were used to investigate the causal effect of the gut microbiome on GD. Through bidirectional two-sample MR analysis, we identified a mutually causal effect between the gut microbiome and GD, which provided evidence supporting the activity of a regulatory TGA.

## Methods

### Study design and the assumption of MR

**Figure 1:**
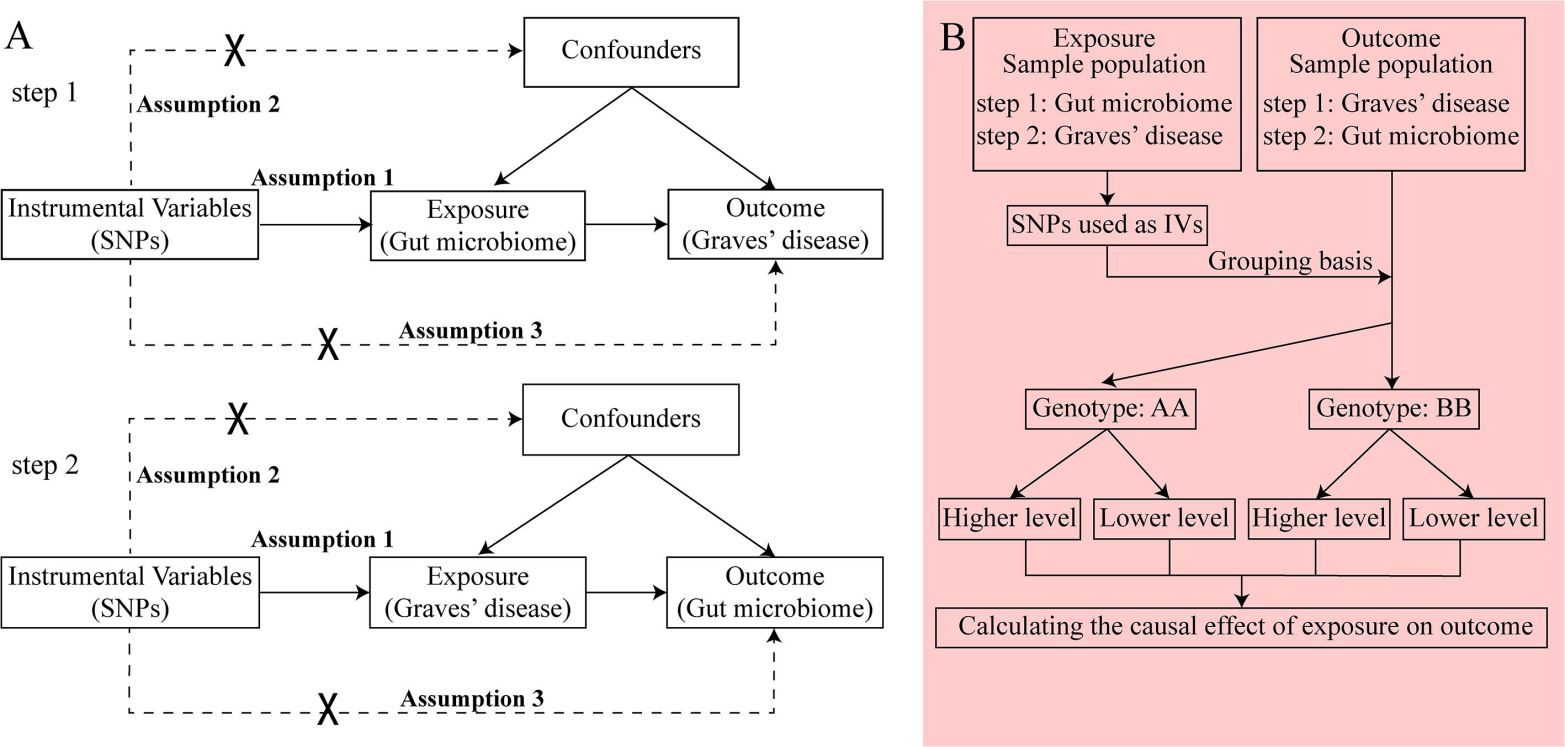
The principle of bidirectional Mendelian randomization (MR) analysis. The process of bidirectional MR analysis was divided into two steps. Each step contained three assumptions (assumptions 1, 2, and 3) and involved a two-sample MR analysis to detect the causal effect between exposure and outcome **(A)**. The idea of a two-sample MR analysis was represented by a flowchart **(B)**. In step 1, the gut microbiome served as exposure, and Graves’ disease (GD) served as the outcome. The single nucleotide polymorphisms (SNPs) obtained from the gut microbiome were considered instrumental variables (IVs), which provided the grouping basis for the GD sample population. The effect of every SNP was combined and the causal effect of gut microbiome on GD was calculated through MR analysis. In step 2, the GD served as exposure, and the gut microbiome served as the outcome. The SNPs obtained from GD were considered IVs, which provided the grouping basis for the gut microbiome sample population. The effect of every SNP was combined, and the causal effect of GD on the gut microbiome was calculated through MR analysis. GD, Graves’ disease; IV, instrumental variable; SNP, single nucleotide polymorphism.

GWAS summary data focusing on the relationships between genetics and disease have been used to detect a causal relationship between the gut microbiome and GD through bidirectional two-sample MR analysis (24, 25). We first selected the gut microbiome as the exposure and GD as the outcome to detect whether the gut microbiome prevents or promotes the occurrence of GD. We also investigated changes in the gut microbiome after the occurrence of GD. The following three assumptions were satisfied for the two-sample MR, according to Bownden et al. (26): (1) the IVs selected from datasets were associated with exposure; (2) the IVs were not associated with any unknown confounders of exposure; and (3) the IVs were associated with outcomes through exposure, but not in other ways (**Figure 1A**). In the process of the MR analysis, the IVs obtained from exposure served to group outcomes of the sample population at conception. Groups with different genotypes represent different exposures. The causal effect of exposure on outcome in each group, which represented the combined effect of each IV (i.e., SNP), was calculated through MR analysis (**Figure 1B**).

### Data sources

All of the data for the present study were obtained from two GWASs, one related to the gut microbiome and one to GD. The GWAS summary data on the gut microbiome were derived from samples comprising 24 cohorts of European, Middle Eastern, East Asian, American Hispanic/Latin, and African American ethnicities, with a total sample number of 18,340 individuals (23). The batch effect was excluded, and details of the 24 cohorts can be found in a previous publication (23). Multiple hypervariable regions (i.e., V1–V2, V3–V4, and V4) in the 16S ribosomal RNA (rRNA) gene were used to investigate the composition of the gut microbiome. Concerning taxonomic profiles, the Ribosomal Database Project (RDP) classifier (v.2.12) was used to submit the reads to SILVA (a reference database) after the samples were rarefied to 10,000 reads by a predefined random seed, and 211 taxa passed taxon inclusion cut-off points (the posterior probability was set as 0.8) including nine phyla, 16 classes, 20 orders, 35 families, and 131 genera. Unknown taxa were excluded from the results (23). The GWAS summary data for GD were obtained from BioBank Japan, which is the largest biobank of the Asian population, containing 212,453 samples (2,176 individuals with GD and 210,277 without), with a dataset of 8,885,805 SNPs (22).

### IV selection

To select the SNPs as IVs that could indicate a potential association between exposure and outcome, we set different thresholds based on differences in exposure. First, we selected the gut microbiome as the exposure. In this case, the IVs were associated with GD at the significance level of *p*< 1 × 10^5^. The minor allele frequency level was set as 0.01. The linkage disequilibrium threshold was set as *R* ^2^ = 0.01, and the distance to search for linkage disequilibrium *R* ^2^-values was set as 500 kb. The super-population was set as East Asian, which was used as the reference panel. If the SNPs selected from exposure were absent in the outcome dataset, the proxy SNPs were significantly related to the selected variants (*R* ^2^ > 0.8). When GD was selected as the exposure, the IVs’ significance level was represented by the genome-wide statistical significance threshold (*p<* 1 × 10^–8^). Moreover, the linkage disequilibrium threshold was set as 0.001, and the clumping window was 10,000 kb. The other parameters were kept constant when the exposure was the gut microbiome. The *F*-statistic of the IVs was calculated to detect whether or not there was a weak IV bias, which was defined as a value of the *F*-statistic< 10 because the bias of the IV estimator was larger than 10% of the bias of the observational estimator, as the *F*-statistic is over 10 (27). IVs were excluded when the *F*-statistic was< 10 using the following formula: *F* = *R* ^2^(*n* – *k* – 1)/*k*(1 – *R* ^2^), where *R* ^2^ is the exposure variance defined by the selected SNPs, *n* is the sample size, and *k* is the number of IVs (28).

### Statistical analysis

Five methods are commonly used to detect the causal effect between exposure and output:

(1) Inverse-variance weighting (IVW): IVW estimates the causal effect of exposure on the outcome by combining the ratio estimates for each SNP, which essentially translates MR estimates into a weighted regression of SNP-outcome effects on SNP-exposure effects (29, 30).
(2) Weighted median: the weighted median method provides unbiased estimates even if up to 50% of the information derives from invalid IVs (31).
(3) Weighted mode: when the largest number of similar individual instrument causal effect estimates comes from valid instruments, the weighted mode method is consistent even if the IVs are invalid (32).
(4) MR-Egger: the MR-Egger is a tool that provides a causal effect through the slope coefficient from Egger regression and also detects small study bias (26).
(5) Simple mode: the simple mode is an unweighted mode of the empirical density function of causal estimation (33).

The characteristics of the five methods are described in **Table 1**. All five methods were used to detect the causal effect between the gut microbiome and GD. The Wald ratio was used to detect the causal effect when there was only one IV obtained from exposure. The causal effect is presented as an odds ratio (OR) when the significance level is less than 0.05, as calculated through the MR method (25).

**Table 1:** Characteristics of five Mendelian randomization (MR) methods.

Method	Description	Number of SNPs	Advantages	Disadvantages
Inverse-variance weighting	Combining the ratio estimates for each SNP, serves as standard method of MR	>1	Simplest and higher effectiveness	Requires all the IVs to be valid
Weighted median	Provides unbiased estimates even if information is from invalid IVs (<50%)	>2	More precise than MR-Egger	Unable to address selection bias
Weighted mode	Calculates the causal effect from valid IVs in the largest cluster	>3	More precise than the simple mode	Smaller power to detect a causal effect than IVW and weighted median
MR-Egger	Provides estimate not affected by the violations of the standard IVs assumptions	>2	Gives consistent estimates even when 100% of genetic variants are invalid IVs	The Instrument Strength Independent of Direct Effect is not valid in some cases
Simple mode	Unweighted mode of empirical density function of causal estimation	>3	Less biased than IVW and weighted median	Less precise than the IVW weighted median

The description column describes the characteristics of each MR method. The pros and cons of the five MR methods were compared to obtain comprehensive results; five MR methods were used in causal effect analysis between the gut microbiome and Graves’ disease (GD). IV, instrumental variable; IVW, inverse-variance weighting; MR, Mendelian randomization; SNP, single nucleotide polymorphism.

In sensitivity analyses, heterogeneity was used to evaluate the compatibility of instrumental variables. Cochran’s *Q* statistics were used to test heterogeneity through the IVW and MR-Egger methods. The effect of heterogeneity should be considered if it existed among IVs (*p*< 0.05) (34). Cochran’s Q was calculated as


$$Q=\sum_{j}w_{j}{\left({\hat{\beta }}_{j}^{IV}-\beta_{j}^{IV}\right)}^{2}$$

where. $\hat{\beta }=\frac{\Sigma_{\text{j}}w_{\text{j}}{\hat{\beta }}_{j}^{IV}}{\Sigma_{\text{j}}w_{\text{j}}}$ and $w_{j}=SE{\left({\hat{\beta }}_{j}^{IV}\right)}^{-2}$ (35). Horizontal pleiotropy indicates that IVs are associated with outcomes through other ways than causal effects, which may lead to false-positive results (*p*< 0.05) (36). Regarding the direct association between the selected IVs and the outcome, horizontal pleiotropy was tested using MR pleiotropy residual sum and outlier (MR- PRESSO). Leave-one-out analysis was performed to identify whether or not a single SNP strongly drives the causal effect of exposure on outcome. To conduct a leave-one-out analysis, harmonized data from exposure and outcome were used as input and tested through the inverse-variance-weighted method. Each SNP from IVs was left out, in turn, to evaluate whether the potential outliers existed, using the TwoSampleMR package (37).

**Figure 2:**
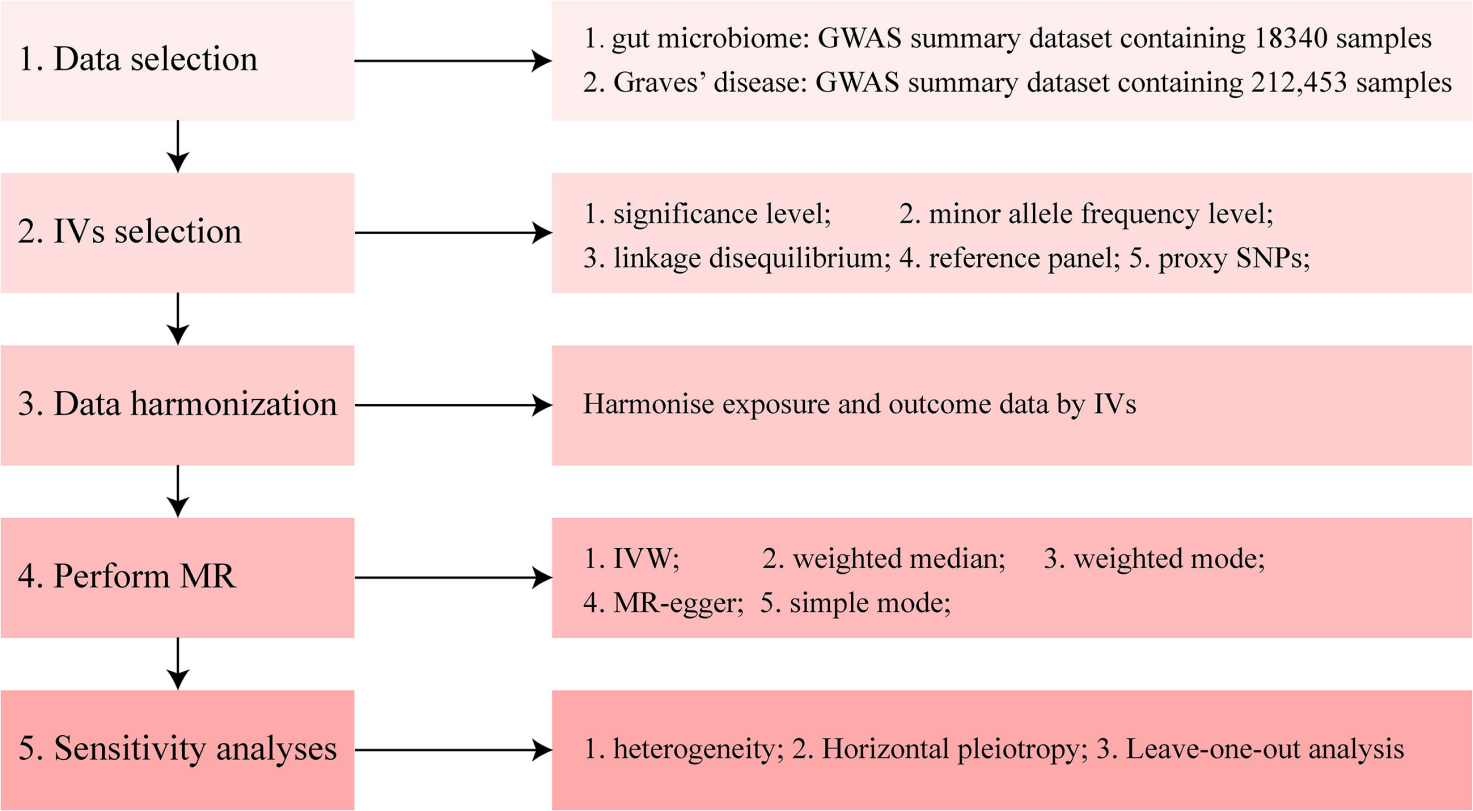
Diagram of Mendelian randomization (MR) analysis processing. There were five main parts of the MR analysis in this study. The workflow was performed twice for when the exposure was gut microbiome and Graves’ disease (GD), respectively. The left boxes indicate the name of the parts, and the right boxes contain the specific content of each part. GWAS, genome-wide association study; IV, instrumental variable; IVW, inverse-variance weighting; MR, Mendelian randomization; SNP, single nucleotide polymorphism.

The methods used in the present study were performed using R software (4.1.0; The R Foundation for Statistical Computing, Vienna, Austria) and the main R packages used in our manuscript include *TwoSampleMR*, *MRPRESSO*, and *MendelianRandomization* (https://github.com/1527311/20221004). The diagram of MR in this study is shown in **Figure 2**.

## Results

### Causal effects of the gut microbiome on patients with GD

To investigate the causal effects of the gut microbiome on GD, we included 1,560 IVs. The *F*-statistic of each SNP was > 10, which indicated that no weak instrument bias existed (**Supplementary material 1**). These IVs were derived from five phyla, 16 classes, one order, 29 families, and 115 genera, and the number of IVs ranged from 1 to 24. We combined the effect of SNPs from the same gut microbiome through MR analysis. Furthermore, the MR method found that two classes, one family, and four genera had a causal effect on GD (**Table 2**).

**Table 2:** Causal effects of the gut microbiome on Graves’ disease (GD).

Exposure	Method	Number of SNPs	*P*-value	OR
Class *Deltaproteobacteria*	MR-Egger	13	0.008	3.603
Class *Deltaproteobacteria*	Weighted median	13	0.018	1.699
Class *Deltaproteobacteria*	Weighted mode	13	0.048	1.791
Class *Mollicutes*	Weighted median	9	0.028	1.655
Class *Mollicutes*	Inverse-variance weighted	9	0.002	1.638
Class *Mollicutes*	Simple mode	9	0.039	2.354
Family *Peptococcaceae*	Inverse-variance weighted	5	0.022	0.536
Genus *Ruminococcus torques* group	Inverse-variance weighted	11	0.031	1.445
Genus *Anaerostipes*	Weighted median	9	0.028	0.489
Genus *Oxalobacter*	Wald ratio	1	0.035	2.395
Genus *Ruminococcaceae* UCG 011	Weighted median	7	0.048	1.379

The exposure represents the specific taxa used for calculating the causal effect between the gut microbiome and GD; the method represents the calculation method used for the Mendelian randomization (MR) analysis in each row; the number of single nucleotide polymorphisms (SNPs) represents the number of instrumental variables (IVs) used for calculations; and the p-values and the odds ratios (ORs) represent the significance and size of the causality of the results, respectively. MR, Mendelian randomization; SNP, single nucleotide polymorphism; OR, odds ratio.

**Figure 3:**
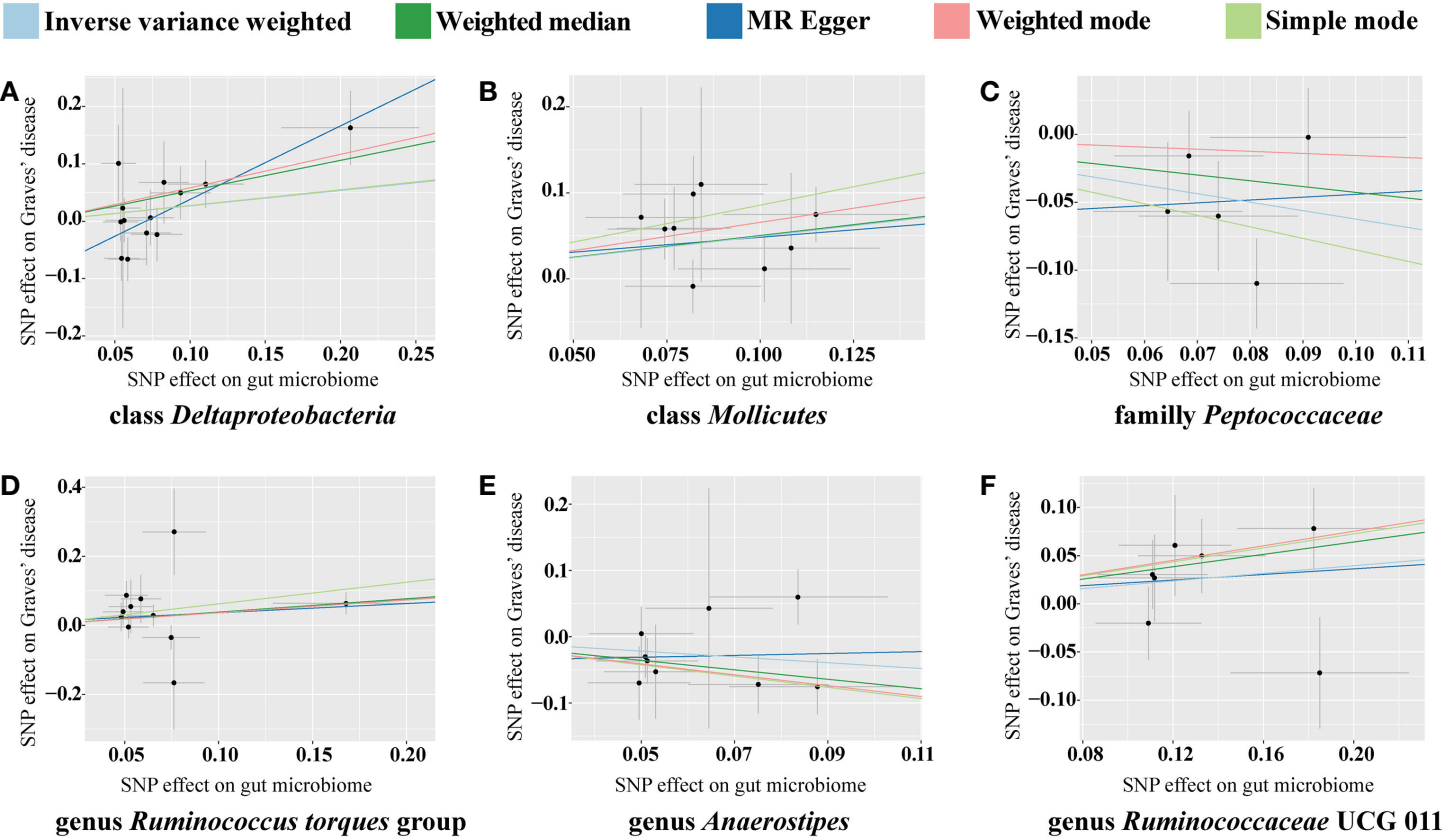
Scatterplot of the effect of the gut microbiome on Graves’ disease (GD). The effect of the gut microbiome on GD is calculated through single nucleotide polymorphisms (SNPs), which provide an association between the gut microbiome and GD through five Mendelian randomization (MR) methods **(A–F)**. In each plot, each dot indicates one SNP from the gut microbiome genome-wide association study (GWAS) summary dataset. The *x*-axis values represent the effect of SNPs on the gut microbiome. The numerical value of the *x*-axis position of each dot equals the absolute value of the β-value of each SNP, and the value of the horizontal error bar equals the standard error of the SNP from the gut microbiome GWAS summary dataset. The *y*-axis values represent the effect of the SNPs on GD. The numerical value of the *y*-axis position of each dot equals the opposite number of β-value of SNP, and the value of the vertical error bar equals the standard error of the SNP from the GD GWAS summary dataset. The β-values and standard errors for SNPs are part of the dataset used to describe the relationship between SNPs and phenotypes; they can be obtained directly by querying the GWAS summary dataset. Different colors of lines represent different MR methods: a causal effect of the gut microbiome on GD was calculated through inverse-variance weighting (IVW) (light blue), weighted median (dark green), MR Egger (dark blue), weighted mode methods (pink), and simple mode (light green). The slope value equals the b-value calculated using the five methods and represents the causal effect of the gut microbiome on GD. The greater the absolute value of the slope, the greater the causal effect. Since the effect allele, an allele to which the effect estimate refers, was identified in the gut microbiome and GD GWAS summary datasets, a positive slope indicates that exposure is a risk factor, whereas a negative slope is the opposite. GD, Graves’ disease; MR, Mendelian randomization; SNP, single nucleotide polymorphism.

The classes *Deltaproteobacteria* and *Mollicutes*, as well as the genera *Ruminococcus torque* group, *Oxalobacter*, and *Ruminococcaceae* UCG 011, were identified as risk factors for GD, and *Deltaproteobacteria* had the largest OR (OR = 3.603) according to the MR-Egger method. The family *Peptococcaceae* and the genus *Anaerostipes* were the protective factors against GD, and *Anaerostipes* had the smallest OR (OR = 0.489) according to the weighted median. The scatterplot, including the causal effect of the gut microbiome on graves' disease, is shown in **Figure 3**.

**Figure 4:**
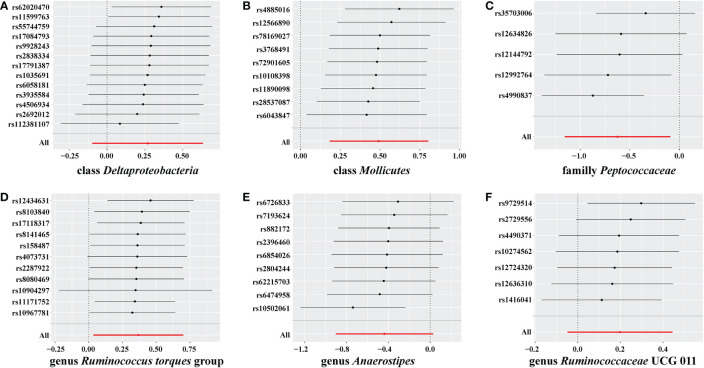
Leave-one-out analysis for gut microbiome on Graves’ disease (GD). The sensitivity of the causal effect of different components of the gut microbiome on GD was analyzed through leave-one-out analysis in **(A–F)**. The error bar represents the 95% confidence interval with the method of inverse-variance weighting (IVW).

In the sensitivity analyses, we performed heterogeneity statistics, horizontal pleiotropy, and leave-one-out analysis. Heterogeneity analysis showed no evidence of a causal effect on GD among the investigated variables of the gut microbiome (**Supplementary material 2**). We used the MR-PRESSO method to detect horizontal pleiotropy, and the results showed that no horizontal pleiotropy existed (**Supplementary material 3**). Leave-one-out analysis showed no SNPs driving the association between the gut microbiome and GD (**Figure 4**).

### Causal effects of GD on the gut microbiome

**Figure 5:**
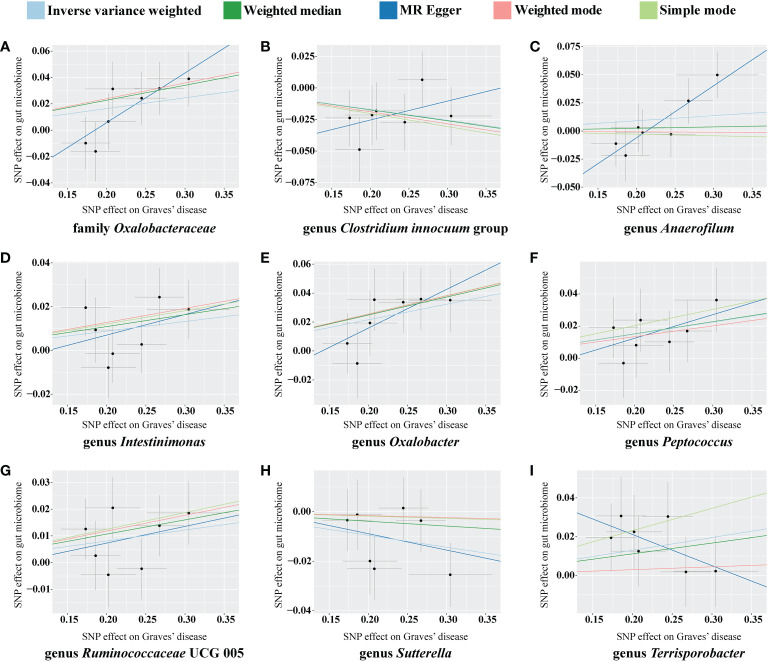
Scatterplot of the effect of Graves’ disease (GD) on the gut microbiome. The effect of the GD on the gut microbiome is calculated through single nucleotide polymorphisms (SNPs), which provide an association between GD and gut microbiome through five Mendelian randomization (MR) methods **(A–I)**. In each plot, each dot indicates an SNP from the GD genome-wide association study (GWAS) summary dataset. The *x*-axis values represent the effect of the SNPs on GD. The numerical value of the *x*-axis position of each dot equals the absolute value of the β-value of each SNP, and the value of the horizontal error bar equals the standard error of SNPs from the GD GWAS summary dataset. The *y*-axis values represent the effect of the SNP on the gut microbiome. The numerical value of the *y*-axis position of each dot equals the opposite number of the β-value of the SNP, and the value of the vertical error bar equals the standard error of SNP from the gut microbiome GWAS summary dataset. The β-values and standard errors for SNPs are part of the dataset used to describe the relationship between SNPs and phenotypes; they can be obtained directly by querying the GWAS summary dataset. Different colors of lines represent different MR methods: a causal effect of GD on microbiome was calculated through inverse-variance weighting (IVW) (light blue), weighted median (dark green), MR-Egger (dark blue), weighted mode methods (pink), and simple mode (light green). The value of the slope equals the b-value calculated through the five methods and represents the causal effect of GD on the gut microbiome. The greater the absolute value of the slope, the greater the causal effect. Since the effect allele, an allele to which the effect estimate refers, was identified in the gut microbiome and the GD GWAS summary dataset, a positive slope indicates that exposure is a risk factor, whereas a negative slope is the opposite.

For the causal effects of GD, 13 SNPs satisfied the criteria. After analysis using the MR method, GD had a causal effect on one family and eight genera (**Figure 5**). Through IVW, the genera *Clostridium innocuum* group (*p* = 0.024, OR = 0.918) and *Sutterella* (*p* = 0.024, OR = 0.953) were down-regulated after the onset of GD. The family *Oxalobacteraceae* and the genera *Anaerofilum*, *Intestinimonas*, *Oxalobacter*, *Peptococcus*, *Ruminococcaceae* UCG 005, and *Terrisporobacter* were up-regulated after the onset of GD (**Table 3**). Of these bacterial communities, the genus *Anaerofilum* had the highest OR (*p* = 0.049, OR = 1.584), which indicated that patients with GD had a higher risk of increased levels of the genus *Anaerofilum*.

**Table 3:** Causal effect of Graves’ disease (GD) on the gut microbiome.

Outcome	Method	Number of SNPs	*P*-value	OR
Family *Oxalobacteraceae*	Weighted median	7	0.007	1.120
Family *Oxalobacteraceae*	Inverse-variance weighted	7	0.015	1.085
Genus *Clostridium innocuum* group	Inverse-variance weighted	7	0.024	0.918
Genus *Anaerofilum*	MR-Egger	7	0.049	1.586
Genus *Intestinimonas*	Inverse-variance weighted	7	0.046	1.045
Genus *Oxalobacter*	Weighted median	7	0.006	1.132
Genus *Oxalobacter*	Inverse-variance weighted	7	0.002	1.114
Genus *Peptococcus*	Inverse-variance weighted	7	0.020	1.078
Genus *Ruminococcaceae* UCG 005	Weighted median	7	0.030	1.055
Genus *Ruminococcaceae* UCG 005	Inverse-variance weighted	7	0.034	1.041
Genus *Sutterella*	Inverse-variance weighted	7	0.024	0.953
Genus *Terrisporobacter*	Inverse-variance weighted	7	0.030	1.067

The outcome represents the specific taxa used for calculating the causal effect between GD and the gut microbiome; the method represents the calculation method used for the Mendelian randomization (MR) analysis in each row; the number of single nucleotide polymorphisms (SNPs) represents the number of instrumental variables (IVs) used for calculations; and the p-values and the ORs represent the significance and size of causality of the results, respectively. MR, Mendelian randomization; SNP, single nucleotide polymorphism; OR, odds ratio.

**Figure 6:**
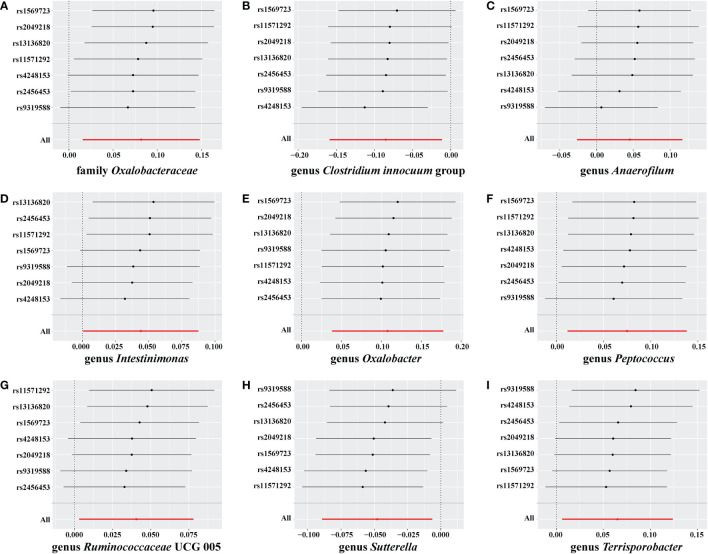
Leave-one-out analysis for Graves’ disease (GD) on the gut microbiome. The sensitivity of the causal effect of GD on a different type of gut microbiome was analyzed using leave-one-out analysis in **(A–I)**. The error bar represents the 95% confidence interval with the method of inverse-variance weighting (IVW).

No weak instrument bias or heterogeneity statistics were identified among the IVs, and no horizontal pleiotropy existed between the IVs and the gut microbiome (**Supplementary material 4**). Leave-one-out analysis revealed that no SNP was significantly associated with the outcome (**Figure 6**).

## Discussion

We conducted a bidirectional MR analysis to identify a causal effect between GD and the gut microbiome. We confirmed an association between GD and the gut microbiome using GWAS summary data, and the results were consistent with previous literature. We found that GD and the gut microbiome reciprocally interacted and that a causal effect existed between GD and the gut microbiome. In addition, risk factors, such as the class *Deltaproteobacteria*, and protective factors, such as the genus *Anaerostipes*, were associated with GD in the gut microbiome. The onset of GD also changed the composition of the gut microbiome; for example, there was an increase in the level of the genus *Anaerofilum* and a reduction in the relative abundance of the genus *Clostridium innocuum*.

The TGA has been well studied and supports an association between the thyroid and the gut (6, 38). The physiological function of and pathological changes in the thyroid are regulated by the function of the gut. On the one hand, the intake of minerals, such as iodine, is necessary for synthesizing thyroid hormones, whereas mineral elements, such as selenium, are essential for maintaining the normal function of the thyroid (39, 40). However, a general association between the intake of essential nutrients through the gut and cell metabolism should be recognized (41). Conversely, the gut interacts with the immune system through nutrients and microbiota, which mediate autoimmune diseases such as GD and thyroid cancer (42). Recent studies on the TGA have mainly focused on the effects of the gut on the thyroid, and few studies have reported on the effects of the thyroid on the gut. The present study found that the levels of the family *Oxalobacteraceae* and genera *Anaerofilum*, *Intestinimonas*, *Oxalobacter*, *Peptococcu*s, *Ruminococcaceae* UCG 005, *Terrisporobacter*, *Clostridium innocuum* species, and *Sutterella* were altered in patients with GD, which revealed a causal effect of GD on changes in the gut microbiome and provided evidence for the reciprocal influence of the thyroid gland on the gut.

Changes in the gut microbiome have long been associated with GD. A prospective clinical study with 39 participants with GD and 17 without GD found that the levels of bacilli, *Lactobacillales*, *Prevotella*, *Megamonas*, and *Veillonella* strains in patients with GD were increased, but that the levels of *Ruminococcus*, *Rikenellaceae*, and *Alistipes* strains were decreased (43). Another study evaluating 55 participants with GD and 48 participants without GD also revealed a change in the gut microbiome, including in the levels of the phylum *Firmicutes*, the phylum *Bacteroidetes*, the family *Prevotellaceae*, the family *Veillonellaceae*, and the genus *Prevotella group* 9 (11). In addition, the treatment of GD also alters the gut microbiome. For example, methimazole up-regulates the levels of *Bifidobacterium* and *Collinsella* but down-regulates the levels of *Prevotella* and *Dialister* (44). Methimazole combined with potential prebiotic berberine achieves a better effect on GD than methimazole alone, and the changes in the gut microbiome include changes in levels of *Lactococcus lactis*, *Enterobacter hormaechei*, and *Chryseobacterium indologenes* (45). The results obtained from patients with GD reveal that changes in the gut microbiome accompany GD, which agrees with our results (45). However, a causal effect between GD and the gut was not illustrated in these previous studies, and the number of samples in these studies ranged from dozens to hundreds, which are not sufficient sample sizes to be representative of the general population. However, in the present study, the data on the gut microbiome contained 18,340 samples from a range of ethnicities, and the GD data contained 212,453 samples of Asian ethnicity, allowing the present study to be more representative of the population.

Potential mechanisms of gut microbiome interactions with GD have recently been investigated. Jiang et al. found that higher levels of *Bacteroides* in GD may alter the intestinal barrier and induce an inflammatory reaction by elevating the concentration of inflammatory factors, which may change the immune status and facilitate the incidence of autoimmune disease (46). In addition, higher thyroid hormone levels have also been positively related to *Lactobacillus* (47). Another study based on a mouse model also reported a causal correlation between the gut microbiome and GD. Researchers have reported that different microbiomes in mice can lead to different presentations of GD and Graves’ ophthalmopathy after injection with a human thyroid-stimulating hormone receptor (*hTSHR*) eukaryotic expression plasmid (48). Vancomycin significantly down-regulates the level of gut microbiota, and decreases the incidence and severity of GD, whereas mice that receive a human fecal material transfer from GD present with increased severity of GD (48). The present study also provides evidence of a causal effect between the gut microbiome and GD based on GWAS summary data from the human population and further found that this causal effect was bidirectional. However, the present study did not identify the specific mechanism for how the gut microbiome interacts with GD.

In the present study, we concentrated on the interactive effects between the gut microbiome and GD through bidirectional MR analysis, which we also used to detect the causal effect between the gut microbiome and GD. Compared with traditional clinical trials, MR analysis allows the identification of sequential relationships as the exposure is defined before the outcome through IVs in the design of the MR analysis, and IVs are less prone to influence by potential confounders (49). The present study utilized *F*-statistics and horizontal pleiotropy, which overcame weak biases and showed a direct association between SNPs and the outcome. Because the data used in the present study were derived mainly from the Asian population, the extrapolation of the results to other ethnic populations should be considered with caution.

In conclusion, the present study confirmed an association and defined a causal effect between GD and the gut microbiome through MR analysis, thereby providing evidence supporting the activity of a TGA in the pathogenesis of GD.

## Data availability statement

The original contributions presented in the study are included in the article/**Supplementary Material**. Further inquiries can be directed to the corresponding authors.

## Author contributions

All authors contributed to the study design. The first draft of the manuscript was written by JC. Data collection and analysis were performed by JC, NW, YL, CM, ZC, CC, and FZ. XQ and WX revised the manuscript and helped with the project administration and funding acquisition. All authors contributed to the article and approved the submitted version.
